# The Role of miR-155 in Antitumor Immunity

**DOI:** 10.3390/cancers14215414

**Published:** 2022-11-03

**Authors:** Katerina Kalkusova, Pavla Taborska, Dmitry Stakheev, Daniel Smrz

**Affiliations:** 1 Department of Immunology, Second Faculty of Medicine, Charles University and Motol University Hospital, 150 06 Prague, Czech Republic; 2 Laboratory of Immunotherapy, Institute of Microbiology of the Czech Academy of Sciences, 142 20 Prague, Czech Republic

**Keywords:** microRNA, immunity, tumors, cancer, immunotherapy, miR-155

## Abstract

**Simple Summary:**

MiR-155 appears to be a significant regulator of immune responses against tumors. The aim of this review is to provide an overview of miR-155 function in distinct immune cell populations and describe how the miR-155-mediated regulation can impact the process of cancer immunoediting. Since miR-155 does not necessarily act only within a single cell, we also touch on the role of miR-155 in cellular communication. Finally, we discuss current developments in the specific targeting of this molecule in the tumor and immune cells and suggest its potential implications in developing novel therapeutic algorithms to increase the efficacy of cancer therapy.

**Abstract:**

MicroRNAs belong to a group of short non-coding RNA molecules that are involved in the regulation of gene expression at multiple levels. Their function was described two decades ago, and, since then, microRNAs have become a rapidly developing field of research. Their participation in the regulation of cellular processes, such as proliferation, apoptosis, cell growth, and migration, made microRNAs attractive for cancer research. Moreover, as a single microRNA can simultaneously target multiple molecules, microRNAs offer a unique advantage in regulating multiple cellular processes in different cell types. Many of these cell types are tumor cells and the cells of the immune system. One of the most studied microRNAs in the context of cancer and the immune system is miR-155. MiR-155 plays a role in modulating innate and adaptive immune mechanisms in distinct immune cell types. As such, miR-155 can be part of the communication between the tumor and immune cells and thus impact the process of tumor immunoediting. Several studies have already revealed its effect on antitumor immune responses, and the targeting of this molecule is increasingly implemented in cancer immunotherapy. In this review, we discuss the current knowledge of miR-155 in the regulation of antitumor immunity and the shaping of the tumor microenvironment, and the plausible implementation of miR-155 targeting in cancer therapy.

## 1. Introduction

The immune system is responsible for maintaining the body’s homeostasis. This maintenance also includes the recognition and elimination of transformed, malignant cells [1]. However, the immune system could be hijacked by the transformed cells and, on the contrary, promote their proliferation and survival in the human body. Many regulatory molecules are known to impact the balance between the pro- and antitumorigenic activities of the immune system. Although many of these molecules are proteins, there are also non-protein molecules, such as non-coding RNAs (ncRNAs), which can substantially contribute to the balance [1,2,3]. One group of ncRNAs is microRNAs (miRNAs). MiRNAs were found to have a substantial regulatory impact on many distinct cellular processes, and research on these molecules has been gaining increasing interest in the last two decades. As miRNAs are often associated with the regulation of important cellular processes, such as apoptosis and proliferation [4,5], which are dysregulated during tumorigenesis, it is no surprise that a large number of studies have been performed in the field of cancer research [6,7]. Nevertheless, despite their direct tumor suppressor or oncogenic functions, miRNAs also participate in tumorigenesis by regulating the immune system [8].

The function of miRNAs is decreasing the messenger RNA (mRNA) levels of protein-coding genes [9]. MiRNAs also participate in the post-transcriptional regulation of many targets, including other regulatory molecules or even transcription factors [10], which make miRNAs powerful players in the biology of the cell. Moreover, their distal transport in extracellular vesicles allows them to be involved not only in the regulation of the single cell in which they are transcribed but also in shaping the transcriptomes of other cells, thereby forming a mode of distal cellular communication [11]. One of the miRNAs participating in all the above-described mechanisms is miR-155, whose role in the tumor immune response is discussed in detail in this review.

## 2. MiRNA Biology

MiRNAs are small (approximately 22 nucleotides), single-stranded ncRNA molecules that participate in the regulation of gene expression at several levels and in cellular communication [12,13]. MiRNAs are evolutionarily conserved among species and have been identified in plants, animals, and even viruses [14,15]. These molecules were discovered almost thirty years ago in *Caenorhabditis elegans*. The first noted miRNA was lin-4, described as a negative regulator of the mRNA for the LIN-14 protein, which is essential for controlling developmental events in *C. elegans* [13,16,17]. The mechanism of its regulatory action was discovered to be based on the complementarity of lin-4 with the LIN-14 mRNA sequence and RNA–RNA interaction [16,17]. One decade after this, the function of these regulatory molecules was recognized. Initially, miRNAs were originally associated with post-transcriptional regulation [18]. However, the functions of miRNAs have been considerably expanded since then, as the mechanisms of their regulatory functions regarding gene expression were found to occur on multiple levels.

**Figure 1:**
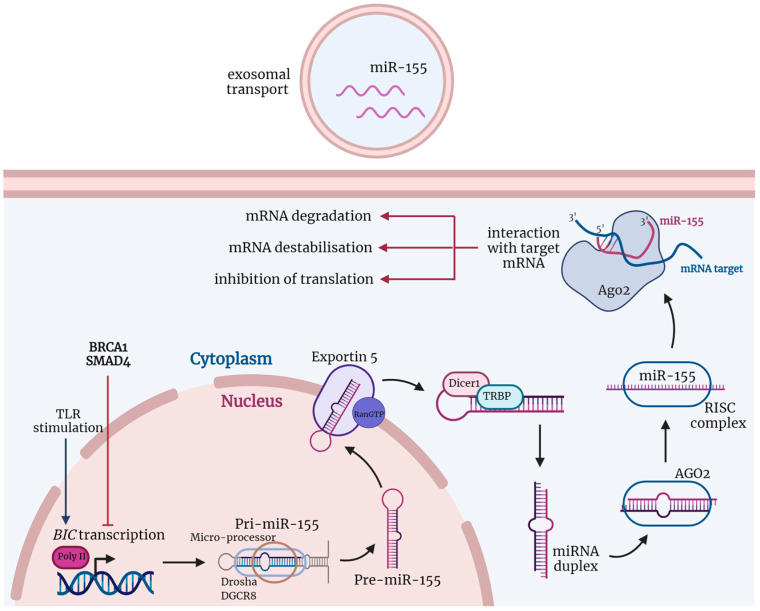
The biogenesis and function of miRNAs are shown via the example of miR-155. The non-coding B cell integration cluster (*BIC*), which encodes miR-155, is transcribed by polymerase II (poly II) into a double-stranded primary RNA transcript (pri-miR-155) after stimuli, such as stimulation of toll-like receptors (TLRs). Pri-miR-155 is cleaved by the ribonuclease—DROSHA and its cofactor binding protein, DGCR8—into precursor miRNA (pre-miR-155). Pre-miR-155 is transported by the GTP-dependent Exportin 5 from the nucleus to the cytoplasm, where the ribonuclease Dicer1 processes it into mature but still double-stranded miR-155. After this, miR-155 and argonaut 2 (AGO2), together with other proteins, create an RNA-induced silencing complex (RISC). RISC unwinds the double-stranded miR-155, and one of its strands is degraded while the second remains and acts as a sequence-specific guide for proteins. MiR-155 in the RISC complex promotes target mRNA degradation and destabilization or inhibits its translation. Created with BioRender.com (agreement number: DV24GT9WQS, accessed on 30 September 2022).

A mature miRNA creates a complex with proteins, namely the ribonucleoprotein (RNP), called the RNA-induced silencing complex (RISC) [19,20,21] (Figure 1). Being part of RNP, miRNA works as a sequence-specific guide that leads RNP to the target sequence. The target sequences are usually mRNAs but may also include other ncRNAs [22]. The miRNA complementarily binds at least seven nucleotides of the target sequence. Longer regions of complementarity are believed to strengthen the binding of the target sequence and miRNA [23]. The interaction of miRNA and target mRNA can result either in the mRNA degradation, its destabilization, or less efficient protein translation. These mechanisms are promoted by the proteins of the RISC complex. Degradation of mRNA is catalyzed by the argonaute protein [19,20], which is usually associated with its binding to the 3′UTR of mRNA [24]. If mRNA is not degraded, its translation can then be controlled on several levels, such as the inhibition of translation initiation, poly (A) shortening, decapping, or altered cap protein binding [25,26].

In contrast to 3′UTR mRNA targeting, binding to the 5′UTR is more often associated with the stabilization of the target mRNA, leading to increased protein translation [27,28]. In addition to mRNA binding, miRNAs can also affect DNA [29]. MiRNAs can even alter the structure of chromatin, which is another mechanism through which miRNAs regulate the expression of their targets [29,30]. The complexity of the gene expression regulation promoted by miRNAs is due to the fact that each miRNA is responsible for the regulation of multiple targets [31]. Moreover, these targets do not have to be transcribed in every cell. Thus, each miRNA can have a different impact on the protein expression in distinct cell types. Additionally, the expression levels of each miRNA differ among distinct cell populations, and they can even differ within a certain cell type depending on the stage of its differentiation. Generally, miRNA-mediated regulation is context dependent.

## 3. MiRNA Biogenesis

MiRNAs can be encoded in the genome as a single unit or they can be clustered. Clustered miRNAs are transcribed together as polycistronic transcripts that are processed into individual mature miRNAs that usually target mRNAs with the related function. MiRNAs are also often coded in introns and intergenic non-coding DNA sequences: “junk DNA” [25,32]. Moreover, their expression can be altered with alternative splicing. A number of miRNAs are classified into families determined by their 5′-end sequence homology [33]. The expression of miRNAs can be tissue specific, and their roles in the regulation of cell processes can differ depending on the cell type [33,34].

The biogenesis of miRNAs is a complex process that begins in the nucleus (Figure 1) [35]. MiRNAs are usually transcribed by RNA polymerase II into double-stranded primary RNA transcripts (pri-miRNAs) with a 5′cap and 3′poly (A) tail. Pri-miRNAs are significantly longer than their mature forms and need to be cleaved by a nuclear microprocessor containing ribonuclease (RNase III) Drosha and its cofactor double-stranded binding protein DiGeorge critical region 8 protein (DGCR8). In the nucleus, pri-miRNA is processed by this microprocessor complex into precursor miRNAs (pre-miRNAs) with a length of 60 to 70 nucleotides [36,37,38]. Thenceforth, pre-miRNAs are transported to the cytoplasm through GTP-dependent protein complex Exportin 5 (XPO5) [39,40]. In the cytoplasm, pre-miRNAs are cleaved and processed into their mature double-stranded miRNAs forms by the RNase III enzyme Dicer [41,42]. However, there are some alternative forms of this miRNA processing pathway [43,44]. The mature, but still double-stranded, miRNAs in the cytoplasm create RISC containing AGO family proteins. One of the miRNA strands is degraded while the second strand remains within RISC [41]. When integrated into the RISC complex, miRNA interacts with complementary RNA and works as a sequence-specific guide for the proteins [45].

## 4. MiRNAs in Tumors and Antitumor Immunity

MiRNA-mediated regulation is important in numerous cellular processes, such as proliferation, differentiation, apoptosis, and metabolism [35]. Dysregulation of miRNAs’ gene expression is involved in several diseases, including cancer. During tumor progression, miRNAs can act similarly as tumor suppressors or oncogenic molecules, depending on their targets [46]. MiRNAs with an oncogenic function are responsible for the downregulation of tumor suppressor genes while miRNAs that act as tumor suppressors are involved in the downregulation of oncogenes and their products [47]. Whereas upregulated levels of oncogenic miRNA are associated with oncogenic diseases, the tumor suppressor miRNA levels are often downregulated during tumorigenesis [48]. For this reason, miRNAs are very often considered to be potential biomarkers in cancer [49]. Moreover, they can also be valuable targets in cancer therapy [50,51,52].

MiRNAs are often transported from cells in vesicles called exosomes. These miRNA-transferring exosomes can be derived from cancer cells or, vice versa, from immune cells [53]. Recently, Wang et al. described exosomal crosstalk between cancer cells and tumor-associated fibroblasts and showed that exosomal miR-155, together with miR-146a, contributed to colorectal carcinoma metastasis [54]. The miR-155 exosomal crosstalk was also described between immune cells, such as dendritic cells [11]. This intercellular communication makes miRNAs a powerful tool in shaping the tumor’s progression and microenvironment [53,55]. Therefore, although research on miRNAs has mostly remained within the field of oncology, these molecules have received considerable attention also in the field of immunology. MiRNAs were found to be involved in the regulation of immune cells [56,57], and since immune cells play a crucial role in antitumor defense mechanisms, a process called immunoediting [58], many miRNA-affecting immune cells are also involved in immunoediting, their involvement in which could be either pro- or antitumorigenic [7,59]. Under some circumstances, miRNAs can even act as immune checkpoint inhibitor molecules [60]. For this reason, all these characteristics make miRNAs an attractive study subject for research on immunoediting in cancer, and one of the currently most studied miRNAs in this field is miR-155.

## 5. MiR-155

MiR-155 is one of the most commonly studied miRNA molecules since its dysregulation is involved in many pathological processes. Its expression levels were shown to control pathways related to essential cellular processes, such as cell proliferation, apoptosis, differentiation, stemness, growth, migration, and angiogenesis [61,62]. Such broad regulatory potential in so many important cellular mechanisms makes miR-155 highly attractive for research and clinical applications [63].

As for genome localization, miR-155 is incorporated within the non-coding B cell integration cluster (*BIC*) gene, which, in humans, is located on chromosome 21. The sequence coding miR-155 is also known as the *MIR155* host gene (*MIR155HG*). The highest expression profile of miR-155 was found in the thymus and spleen, and increased levels of miR-155 were also found in CD34^+^ hematopoietic stem cells [64]. This finding led to the hypothesis that miR-155 could inhibit the differentiation of these cells [65]. The same study also suggested that a number of molecules participating in the hematopoietic differentiation are targets of miR-155 as their 3′UTR of mRNA includes the binding side for this miRNA. Similar miR-155 expression patterns were reported during erythropoiesis, where high expression levels of miR-155 were associated with lower differentiation states of erythroid cells, whereas their mature counterparts had these levels significantly decreased [66]. In addition, miR-155 plays a role in lymphocyte development [67,68]. Generally, miR-155 has been shown to play important roles in the regulation of myelopoiesis, lymphopoiesis, and erythropoiesis [65,69], and its dysregulation can negatively impact the development of cells derived from these lineages [70]. Recent studies also showed that miR-155 promotes the proliferation of tumor cells and inhibits their apoptosis through PTEN/PI3K/AKT targeting [62,71]. In addition, this mechanism was reported even upon cellular crosstalk when miR-155 was transferred in exosomes [71].

The function of miR-155 can be explained by its target genes. MiR-155 expression is regulated by several proteins that usually directly bind the promoter region of *BIC*. The tumor suppressor protein BRCA1 was described to negatively regulate the expression of miR-155 via inducing the deacetylation of histones on the miR-155 promoter [72]. Another transcriptional regulator of miR-155 is SMAD family member gene 4 (SMAD4), which can cause the repression of miR-155 expression as a result of TGF-β1 signaling [73]. NF-κB and TP53 were also shown to regulate miR-155 expression by directly interacting with the promoter [74,75]. Regulation of miR-155 levels does not necessarily always rely on interaction with the *BIC* promoter. The reduction of miR-155 levels can also be elicited post-transcriptionally by decreasing the stability of the miR-155 precursor and its maturation. An example of the negative regulation of miR-155 on the post-transcriptional level could be represented by the anti-inflammatory cytokine IL-10 [76,77].

MiR-155 is mostly considered an oncogenic miRNA in the field of oncology. In many studies, its upregulation was found to correlate with tumor occurrence and often with poor prognosis for many cancers [78,79]. Since miR-155 plays a role in the primary differentiation of myeloid progenitors, it is no surprise that its oncogenic property was mostly revealed in hematologic malignancies [80,81]. In addition, its oncogenic potential was also described in solid tumors, such as breast cancer [79,82,83] or gliomas [84].

## 6. MiR-155 in Antitumor Immunity

**Figure 2:**
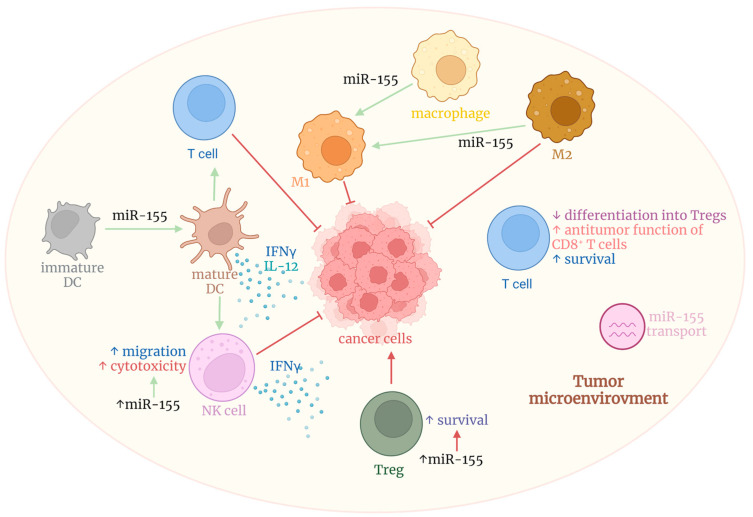
MiR-155-mediated shaping of the immune antitumor response. Created with BioRender.com (agreement number: RT24GT9RIE, accessed on 30 September 2022).

MiR-155 significantly impacts immune cells by targeting important regulatory molecules and transcriptional factors that regulate the immune system (Figure 2). Generally, the miR-155 expression increases after immune cell activation [85,86].

### 6.1. Danger Signals

MiR-155 levels increase in macrophages and dendritic cells after their response to danger signals: pathogen-associated molecular pattern (PAMP) or damage-associated molecular pattern (DAMP) molecules [86,87,88,89]. Several PAMP and DAMP molecules have been described in connection with miR-155 upregulation, such as lipopolysaccharide (LPS), polyinosinic–polycytidylic acid (poly I:C), and interferon beta (INF-β) [86,88,90]. This upregulation can be mediated by NF-κB signaling [91] or the loss of Notch signaling [92]. High levels of miR-155 maintain the activated phenotypes of immune cells by inhibiting the expression of negative immune regulators, including inositol-polyphosphate-5-phosphatase-1 (SHIP-1) [93], and also contribute to the increased production of pro-inflammatory cytokines, such as TNF-α and IL-1β [93].

### 6.2. Dendritic Cells

Dendritic cells (DCs) are professional antigen-presenting cells and linkers of innate and adaptive immunity, which is accomplished via their unique ability to induce naïve T cell activation [94]. Monocyte-derived DCs upregulate miR-155 during their maturation, which is suggested to be part of a negative feedback loop, since miR-155 targets the toll-like receptor and IL-1 inflammatory pathways [95]. Increased levels of miR-155 are also associated with the enhanced ability of DCs to induce T cell proliferation [96]. DCs lacking miR-155 display an antigen presentation deficiency, downregulated production of IL-12, and decreased expression of the chemokine receptor CCR7. Hence, these DCs display deficiencies in their maturation, migration, and ability to induce T cell activation [67,97,98,99]. MiR-155 targeting c-Fos was suggested to be the mechanism mediating DC maturation [100]. However, other miR-155 targets, such as the suppressor of cytokine signaling-1 (SOCS-1), arginase 1, and Jarid2, can contribute to these mechanisms as well [67,97,98,99].

DCs can amplify their functions through mutual communication, which is mainly promoted by exosomes. Functional microRNAs can be transferred in exosomes (nanovesicles) and thus influence the phenotype of the acceptor DC. Therefore, mature DCs can even affect the maturation phenotypes of other acceptor DCs [101]. Overall, the miR-155-induced maturation of DCs is associated with their antitumor phenotype, as it leads to the production of pro-inflammatory cytokines.

The most important regulatory role of miR-155 in DC functionality was further confirmed in other studies, both in vitro and in vivo. The in vitro studies showed that DCs treated with miR-155-enriched exosomes produced more IL-12 and IFN-γ, the cytokines that significantly contribute to the DCs’ antitumor activities [102]. These exosome-treated DCs were found to be superior in inducing the differentiation, proliferation, and cytotoxicity of T cells [103]. Moreover, in vivo studies in a mouse model of colorectal cancer showed that adoptively transferred miR-155-treated DCs promoted increased tumor infiltration with cytotoxic and helper lymphocytes, whereas infiltration with regulatory lymphocytes was decreased [103]. These findings show that miR-155 in DCs appears to act in an antitumorigenic manner.

### 6.3. Macrophages

Macrophages are thought to be the most common myeloid cells present in the tumor microenvironment. Herein, the macrophages can display either a pro-tumorigenic or antitumorigenic role. Pro-tumorigenic (M2) macrophages support the growth of the primary tumor and the spread of metastases. The correlation between their abundance in the tumor and prognosis has been described for many cancers [104,105,106]. Antitumorigenic (M1) macrophages, on the other hand, can promote infiltration with cytotoxic lymphocytes or the antitumor activities of the tumor-infiltrating DCs [107]. MiR-155 was found to directly target and downregulate the phosphatase SHIP-1. The overexpression of miR-155 in macrophages was shown to repress SHIP-1 activity, leading to enhanced AKT signaling in stimulated cells [108]. In addition, the overexpression of miR-155 in tumor-associated macrophages (TAMs) led to macrophages’ re-polarization into pro-inflammatory (antitumorigenic) M1 macrophages [109]. However, these data contrast with the role of SHIP-1 in macrophage differentiation because SHIP-1-deficient mice were found to profoundly skew macrophage differentiation into the M2 phenotype [110]. However, regardless of the role of SHIP-1, genetic miR-155 overexpression was demonstrated to promote the generation of M1-skewed TAMs [109].

Tumor-infiltrating macrophages are also frequent producers of exosomal miRNAs, which significantly shape the cellular crosstalk in the tumor microenvironment [111]. This mechanism is also used by tumors, as many tumor cells are also producers of miR-155, therefore impacting the immune cells in the tumor milieu [112,113].

### 6.4. Myeloid-Derived Suppressor Cells

MiR-155 impacts the functions of myeloid-derived suppressor cells (MDSCs), a population of immature myeloid cells with an immunosuppressive function [114]. The presence of MDSCs in the tumor microenvironment is often associated with worse antitumor immune responses and disease prognosis [115]. Loss of miR-155 in MDCSs was found to increase their migration and immunosuppressive potential [116]. This potential can even override the tumor growth inhibition mediated by the loss of miR-155 [117]. This override was demonstrated by a finding that showed that although the inhibition of miR-155 in the tumor cells suppressed their growth, the loss of miR-155 in the tumor microenvironment promoted tumor growth [117]. In contrast, the study by Li et al. suggested that the loss of miR-155 could be associated with worse MDSC proliferation by showing that the upregulation of miR-155 was linked to enhanced MDCS expansion [118]. Chen et al. also revealed that the loss of miR-155 was associated with the reduced infiltration of MDSCs into the tumor microenvironment [119]. Mechanistically, miR-155 was suggested to contribute to the MDSC suppressor activity in two ways: by inhibiting SOCS-1 and weakening the capacity of MDSCs to induce regulatory T cells (Tregs) [119]. However, the role of miR-155 in MDSCs is ambiguous, since miR-155 produced by leukemia was found to induce MDSCs and enhance their function [120].

### 6.5. Natural Killer Cells

Another important role in miR-155-mediated antitumor immunity is played by natural killer (NK) cells. The number of NK cells is often associated with favorable clinical outcomes of the disease [121,122]. NK cells are also regulated by miR-155. The miR-155 levels in NK cells were found to be elevated after their stimulation and associated with the production of IFN-γ, which was mechanistically promoted by the miR-155-mediated inhibition of SHIP-1 [123]. The miR-155-mediated inhibition of SHIP-1 was also found to regulate NK cell chemotaxis and migration. An miR-155 deficiency followed by SHIP-1 overexpression resulted in impaired F-actin cytoskeleton polymerization, which then negatively impacted tumor infiltration with NK cells [124]. In addition, increased levels of miR-155 were found to be associated with enhanced cytotoxicity of NK cells towards tumors [125]. Taken together, these findings indicate that miR-155 may be a positive regulator of NK cells in antitumor immunity and suggest an ability to migrate into the tumor microenvironment.

### 6.6. T Cells and Immune Checkpoint Inhibitors

Very early, in 2002, Haasch et al. found that miR-155 expression was significantly increased in CD4^+^ T cells after their activation [85]. Subsequent studies showed that this miRNA plays a crucial role in the proliferation and differentiation of lymphocytes [126,127]. MiR-155 was also found to promote CD4^+^ T cell differentiation into the Th17 phenotype [67,128], and this differentiation was induced by STAT3 [129]. Vice versa, CD4^+^ T cell differentiation into the Th2 phenotype is suppressed by the miR-155-mediated downregulation of c-Maf, which is a Th2 response-promoting transcription factor and an inhibitor of the Th1 response [130]. Therefore, miR-155 promotes the Th1 differentiation of CD4^+^ T cells [131,132].

MiR-155 also affects the function of Tregs. Tregs play an essential role in tumor immune escape as they promote a pro-tumorigenic environment via cytokine production and cell–cell communication [133,134]. MiR-155 is highly expressed in Tregs, and its decrease leads to a reduction in their numbers [135]. The Treg-specific transcriptional factor, FoxP3, also regulates BIC’s transcription [136,137,138]. It was further demonstrated that miR-155 could enhance CD4^+^ T cell differentiation towards Tregs [139] and that reduced miR-155 levels in Tregs can shorten their survival [136]. However, contrasting data were obtained with an miR-155 inhibitor (antagomir), which was found to modulate the balance between Tregs and Th17 via the Jarid2/Wnt/β-catenin pathway. The miR-155 inhibitor enhanced Tregs in vitro whereas Th17 cells were decreased [140]. Although contrasting, these findings point to the fact that miR-155 can significantly impact the functionality of Tregs and thus shape their role in antitumor immunity.

CD8^+^ T cells are critical effector cells of antitumor activities in the immune system [141]. These cells were found to be much less efficient in tumor growth control in the absence of miR-155 expression [142]. On the other hand, increased expression of miR-155 promoted their antitumor activity. The mechanism behind this regulation of CD8^+^ T cell effector function was based on the miR-155 targeting of SOCS-1 [142].

Immune checkpoint inhibitors such as cytotoxic T lymphocyte-associated antigen-4 (CTLA-4), programmed cell death protein 1 (PD-1), and its ligands PDL-1 and PDL-2 participate in the inhibition of T cell activation [143]. Inhibitors of these molecules enhance antitumor immune responses and are widely used in the current cancer immunotherapy approach, which is considered to be one of the most successful cancer immunotherapy strategies of the past decade [144]. The expression of these inhibitors can also be regulated by miR-155, as demonstrated in a study where the direct binding of miR-155 to the 3′UTR of PDL-1 mRNA downregulated the expression of this critical regulator of T cell function [145].

## 7. MiR-155 in Cancer Immunotherapy

Linking the above-described characteristics of miR-155 together, it is undoubtable that this molecule has potential clinical relevance in cancer therapy, especially in immunotherapy. There are many potential options for the usage of miR-155 in different immunotherapeutic modalities.

One of the most promising approaches relates to active cellular immunotherapy based on DCs. In these cells, miR-155 was shown to improve the efficacy of DC-based cancer vaccines in a mouse model. In this study, miR-155 overexpression in DCs enhanced their ability to increase CD8^+^ T cell antitumor responses [96]. In vivo, the proteasome inhibitor Bortezomib, approved for multiple myeloma treatments, was also found, in addition to other mechanisms, to induce the miR-155-mediated downregulation of SOCS-1 and SHIP-1. Suppression of these immune system regulators in the final stages led to the suppression of PD-1-mediated T cell exhaustion [146]. Another in vivo study showed that miR-155 derived from breast cancer cells was recently described to enhance the recruitment of antitumor immune cells, as the repression of SOCS-1 is associated with the upregulation of several chemokines. Moreover, miR-155 was reported to change a “cold tumor” into a “hot one” and thus sensitize the tumor for checkpoint blockage immunotherapy [147]. Bioinformatics analysis also revealed that higher levels of miR-155 in tumors were correlated with an enhanced antitumor immune profile and favorable outcomes. Moreover, the study also revealed that high miR-155 levels in serum were a good prognostic marker for breast cancer. The authors of this study suggested that increasing the levels of miR-155 in breast tumors could improve the efficacy of cancer immunotherapy by increasing tumor infiltration with immune cells [147]. Indeed, the potential of miR-155 as a predictive biomarker for immunotherapeutic efficacy has already been reported [148]. The mechanisms involved in this process are presumably based on the potential of miR-155 to modify the tumor microenvironment, namely the immune cells within. An example of this modification was demonstrated by the repolarization of the tumor-infiltrating macrophages (TAMs) [149]. This study showed that miR-155-overexpressor redox/pH dual-responsive hybrid polypeptide nanovectors that targeted TAMs repolarized immunosuppressive macrophages into M1 macrophages with antitumor properties. These properties were displayed via robust tumor regression, which was also associated with increased T and NK cell activation in the regressing tumors [149].

On the other hand, another study suggested that miR-155 should be therapeutically downregulated because high levels of miR-155 were found to be associated with cancer resistance to chemotherapy and radiotherapy [150]. Therefore, under these circumstances, miR-155 inhibitors might seem to represent a novel therapeutic approach to sensitizing tumors to chemotherapy or radiotherapy [150]. This avenue is tempting and might appear promising, but, as demonstrated by other studies discussed above, the performance of miR-155 in tumors can be ambivalent, and the flat downregulation of miR-155 can trigger multiple mechanisms with different impacts on the disease state. Downregulation of miR-155 could indeed sensitize tumors to chemotherapy or radiotherapy on the one hand, but, on the other, this intervention could remodel the tumor’s immune microenvironment, and the tumor could become resistant to the immune system and immunotherapy. The outcome of this flat intervention could finally be the chemo/radiotherapy-induced disappearance of large masses of the tumor while simultaneously rendering the remaining (surviving) tumor mass more resistant to the immune system and immunotherapy, which could, in the end, cause a disease relapse.

## 8. Future Perspectives

A large number of studies related to the field of cancer research have shown the oncogenic nature of miR-155 [83,151]. Being upregulated in many cancers and promoting the stemness of cancer cells, which is strongly associated with poor prognosis, and increasing their resistance to chemotherapy and radiotherapy [150], the systematic downregulation of miR-155 was initially a convenient therapeutic approach. However, being deeply immersed in immune system regulation and participating in the antitumor response, mainly by affecting many distinct immune cell populations, the harvesting of the regulatory powers of miR-155 for cancer therapy needs to be reconsidered based on the implementation of novel therapeutic algorithms. These algorithms should stem not only from the disease severity, including the tumor staging and grading, but also from the tumoral/paratumoral immune signatures inflicted by the disease or its treatment [152,153,154,155,156]. Guided by these signatures, the design of the therapeutic algorithm could then even include both miR-155 downregulation and upregulation, each corresponding to the time of treatment and the type of therapeutic modality. For instance, intratumoral miR-155 downregulation could be initially indicated as a neoadjuvant therapy before chemotherapy to sensitize the tumor to chemotherapy or radiotherapy [150]. Then, following chemotherapy or radiotherapy, miR-155 upregulation in a specific immune cell population, such as tumor-infiltrating macrophages [149], could become the basis of adjuvant immunotherapy.

However, these algorithms will presumably require the precise targeting of miR-155 in specific cell types, namely the immune cell populations. This precision targeting could rely on novel technologies, such as nanovectors or adoptive cellular immunotherapy, where individual immune cell types could be produced ex vivo and modified accordingly. Such differential miR-155 targeting could be a promising avenue to enhance antitumor immunity and the efficacy of cancer therapy.

## 9. Conclusions

MiR-155 is involved in the regulation of immunoediting processes on many levels, participates in changes in cancer cells and immune cells, and plays a role in their crosstalk, and its expression levels can significantly shape the severity and therapeutic resistance of the patient’s disease. These characteristics make miR-155 a potential target in cancer diagnostics, prognostics, and treatment. This review showed the promising implications of miR-155 targeting in cancer therapy. With an increasing understanding of miR-155′s functions in both cancer and immune cells and its precision targeting using newly developing technologies, miR-155 could become an immune checkpoint molecule of interest. As such, its targeting could be implemented in novel algorithms of cancer therapy in the near future.
